# Youth violence: a novel initiative to decrease morbidity and mortality from penetrating trauma

**DOI:** 10.1186/1757-7241-21-S1-S31

**Published:** 2013-05-28

**Authors:** N Rhead, S Jackson, C Neary-Bremer, S White, D Lockey

**Affiliations:** 1 Street Doctors. Liverpool. UK

## Background

Interpersonal violence is the third leading cause of death in Europe in 15 to 29 year olds, accounting for 14,899 fatalities.[1] In Liverpool UK, a novel prevention and intervention training scheme has been established to decrease morbidity and mortality amongst young people. Medical Students provide high risk young people with the skills, knowledge and confidence to manage victims in the minutes immediately following injury concentrating on haemorrhage control.

## Aim

To provide young high risk groups with the skills, knowledge and confidence to use basic haemorrhage control techniques for penetrating trauma.

## Method

Young offenders are known to be at risk of witnessing penetrating trauma in the community.[2] They are often present at the scene of violent incidents but few possess the skills to act positively and assist the victim. The training program has been expanded to London, Manchester, Nottingham and Sheffield and delivered more than 1,400 young offenders. Following a training session 21 young offenders in Liverpool were asked to complete a survey.

## Results

50% of the sample group had witnessed penetrating trauma in the community prior to the course. 90% believed that basic haemorrhage control teaching is necessary for young people of Liverpool. Furthermore 90% agreed that they were more likely to assist a friend should they become a victim of penetrating trauma and that they would feel more confident managing a victim following the teaching course. All of the participants felt that they were more aware of the impact to health that being shot or stabbed can have upon victims following teaching courses.

## Discussion & conclusion

Violence amongst young populations is a growing public health problem.[3,4] Training health care professionals provides better outcomes from penetrating traumas but they rarely witness such incidents. This project suggests that many high risk young people witness penetrating trauma but are unable to deliver positive interventions to aid the victim. The training sessions developed by Street Doctors are a cost effective method of providing trained personnel at the scenes of violent incidents who can act immediately to deliver basic haemorrhage control and achieve haemostasis promptly. By bridging this gap in treatment it is hoped that outcomes from major penetrating trauma can be improved.

## References

[B1] The global burden of disease: 2004 update 2008 Geneva, World Health Organization http://www.who.int/healthinfo/global_burden_disease/2004_report_update/en/index.html accessed 17 August 2010

[B2] Brooke BS Patterns and outcomes among penetrating trauma recidivists: it only gets worse Journal of Trauma-Injury Infection & Critical Care 2006 16832245 10.1097/01.ta.0000224143.15498.bb

[B3] Crewdson K Lockey D Weaver A Davies G Is the prevalence of deliberate penetrating trauma increasing in London? Experiences of an urban pre-hospital trauma service Injury 2009 4 3 240 244 19232594 10.1016/j.injury.2008.10.008

[B4] Sethi D European Report on Preventing Violence and Knife Crime among Young People 2010 WHO Europe 1 102

